# Systemic-Immune-Inflammation Index as a Promising Biomarker for Predicting Perioperative Ischemic Stroke in Older Patients Who Underwent Non-cardiac Surgery

**DOI:** 10.3389/fnagi.2022.865244

**Published:** 2022-04-01

**Authors:** Faqiang Zhang, Mu Niu, Long Wang, Yanhong Liu, Likai Shi, Jiangbei Cao, Weidong Mi, Yulong Ma, Jing Liu

**Affiliations:** ^1^Anesthesia and Operation Center, The First Medical Center, Chinese PLA General Hospital, Beijing, China; ^2^Department of Neurology, The Affiliated Hospital of Xuzhou Medical University, Xuzhou Medical University, Xuzhou, China; ^3^Department of Pain Medicine, The First Medical Center, Chinese PLA General Hospital, Beijing, China

**Keywords:** systemic-immune-inflammation index (SII), perioperative stroke, postoperative complication, inflammation, older patients, biomarker

## Abstract

**Objective:**

This study aimed to investigate the clinical prognostic values of the preoperative systemic-immune-inflammation index (SII) in older patients undergoing non-cardiac surgery, using perioperative ischemic stroke as the primary outcome.

**Methods:**

This retrospective cohort study included older patients who underwent non-cardiac surgery between January 2008 and August 2019. The patients were divided into SII < 583 and SII ≥ 583 group according to the optimal SII cut-off value. The outcome of interest was ischemic stroke within 30 days after surgery. Primary, sensitivity, and subgroup analyses were performed to confirm that preoperative SII qualifies as a promising, independent prognostic indicator. Propensity score matching (PSM) analysis was further applied to address the potential residual confounding effect of covariates to examine the robustness of our results.

**Results:**

Among the 40,670 included patients with a median age of 70 years (interquartile range: 67, 74), 237 (0.58%) experienced an ischemic stroke within 30 days after surgery. SII ≥ 583 was associated with an increased risk of perioperative ischemic stroke in multivariate regression analysis [odds ratio (OR), 1.843; 95% confidence interval (CI), 1.369–2.480; *P* < 0.001]. After PSM adjustment, all covariates were well balanced between the two groups. The correlation between the SII and perioperative ischemic stroke remained significantly robust (OR: 2.195; 95% CI: 1.574–3.106; *P* < 0.001) in the PSM analysis.

**Conclusion:**

Preoperative SII, which includes neutrophil, platelet, and lymphocyte counts obtained from routine blood analysis, was a potential prognostic biomarker for predicting perioperative ischemic stroke after non-cardiac surgery in older patients. An elevated SII, based on an optimal cut-off value of 583, was an independent risk factor for perioperative ischemic stroke.

## Introduction

With the rapidly aging population worldwide, the number of older patients undergoing surgery and general anesthesia has increased substantially. Given the advanced age or broad range of comorbidities, older adults are more vulnerable to postoperative complications (Handforth et al., 2015; Marcantonio, 2017). Perioperative ischemic stroke is an uncommon but lethal procedure-related complication, in which nearly half of all patients die within 10 years (Zhang et al., 2022). Due to the initially “silent” signs or symptoms and the delay to diagnostic imaging, perioperative ischemic stroke is not only difficult to identify, but, when finally diagnosed, it is also more likely for patients to have missed the narrow time window for safe treatment with tissue-type plasminogen activator thrombolysis (Mao et al., 2017). Therefore, the search for preoperative biomarkers is an urgent and challenging task in clinical practice. These biomarkers would allow for better population stratification and optimal allocation of healthcare resources.

Inflammation is intimately associated with the occurrence, progression, and prognosis of ischemic stroke (Macrez et al., 2011). In animal models of brain ischemia, brain-infiltrating innate and adaptive immune cells contribute to ischemic cerebral damage and exacerbate neurological deficits (Mracsko et al., 2014). In addition, the inflammatory response results in activation of coagulation, in turn, coagulation factors potentiate inflammatory reactions (Redecha et al., 2007). Furthermore, preoperative chronic inflammation, characterized by the aberrant accumulation of inflammatory cells and inflammatory factors, increases postoperative cardiovascular complications and all-cause mortality (Ettinger et al., 2015; Fragiadakis et al., 2015). Systemic inflammation levels can be evaluated and quantified using a variety of hematological markers routinely measured in the clinical settings or the indices calculated from these measurements (Nøst et al., 2021). Neutrophil-to-lymphocyte ratio (NLR), platelet-to-lymphocyte ratio (PLR), C-reactive protein (CRP), and Glasgow prognostic score (GPS) are closely associated with postoperative outcomes in patients who undergo tumor resection (Jomrich et al., 2021). Systemic-immune-inflammation index (SII) (Hu et al., 2014), derived from neutrophil, lymphocyte, and platelet counts, has not been assessed or applied to predict perioperative ischemic stroke in older patients undergoing non-cardiac surgery.

In this study, we examined the prognostic value of the preoperative SII in older patients undergoing non-cardiac surgery. We hypothesized that an elevated SII could indicate an increased risk of perioperative ischemic stroke. Herein, we performed a large retrospective cohort study of older patients undergoing non-cardiac surgery to investigate the association between preoperative SII and perioperative ischemic stroke.

## Materials and Methods

The study protocol was reviewed and approved by the institutional ethics committee of Chinese PLA General Hospital (No. S2021-135-01), and the need for informed content was exempted. This manuscript adhered to the applicable guidelines as presented in the Strengthening the Reporting of Observational Studies in Epidemiology statement.

### Inclusion and Exclusion Criteria

**FIGURE 1:**
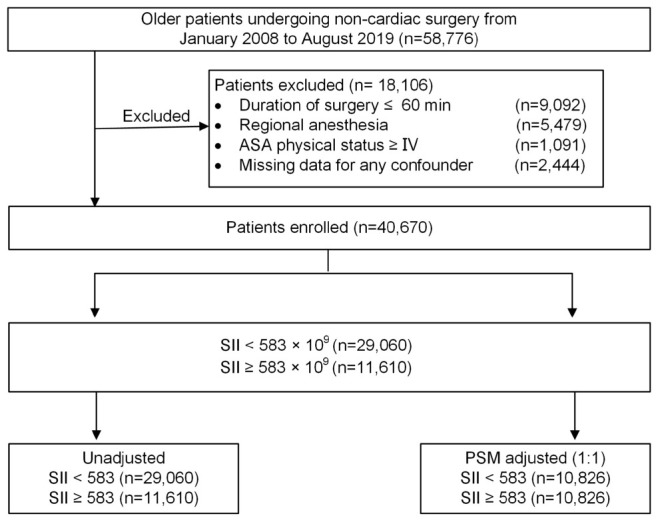
Study profile. ASA, American Society of Anesthesiologists; PSM, propensity score matching.

Patients who underwent non-cardiac surgery between January 2008 and August 2019 at Chinese PLA General Hospital were initially screened from a perioperative retrospective database. The inclusion criteria were as follows: (1) aged 65 yr or older; (2) underwent non-cardiac surgery; (3) received general anesthesia; and (4) were with duration of surgery >60 min. Patients who presented with an American Society of Anesthesiologists (ASA) classification of ≥IV, were performed under regional anesthesia, or had missing clinical data were excluded. Among patients who underwent multiple surgeries during the study period, only the first eligible surgery was considered. A flow diagram of the patient selection process is displayed in Figure 1.

### Clinical Outcome

The primary outcome of interest was perioperative ischemic stroke, defined as an episode of neurological dysfunction, such as motor, sensory, or cognitive dysfunction, caused by focal cerebral, spinal, or retinal infarction within 30 postoperative days (Sacco et al., 2013). Diagnoses of stroke are confirmed by a combination of neuroimaging and clinical evidence of cerebrovascular ischemia during hospital stay. In our study, perioperative ischemic stroke patients were identified if discharge records included at least 1 ICD-9-CM/ICD-10-CM diagnosis code for stroke (Supplementary Table 1).

### Definition of Variables and Data Collection

Serum concentrations of preoperative peripheral platelet, neutrophil, and lymphocyte were the most recent blood counts measured within 3 days prior to surgery. The SII, NLR, and PLR were calculated as follows: SII = platelet × neutrophil/lymphocyte, NLR = neutrophil/lymphocyte, PLR = platelet/lymphocyte. In order to visualize perioperative ischemic stroke in relation to SII, NLR, and PLR, parameters were grouped into high and low by the optimal cut-off points using the receiver operating characteristic curve (ROC).

The exposure of interest was preoperative SII. Result from the ROC analysis indicated that the optimal cut-off value for the SII was set at 583 × 10^9^ for predicting perioperative ischemic stroke. Consequently, the SII was stratified into low (< 583 × 10^9^) and high (≥ 583 × 10^9^) for subsequent analyses.

Preoperative covariates of interest, such as age, sex, body mass index (BMI), ASA classification, hypertension, diabetes mellitus, prior ischemic stroke, coronary heart disease, arterial fibrillation, peripheral vascular disease, renal dysfunction, and preoperative use of β-blockers or aspirin, were noted. The indices derived from the preoperative laboratory data, including hemoglobin, albumin, total bilirubin, prothrombin time, were defined as the most recent blood counts measured within 3 days prior to surgery. In addition, we collected surgical and anesthetic information, such as preoperative mean arterial pressure (MAP), surgical procedures, duration of procedures, estimated blood loss, intraoperative hypotension (MAP ≤ 65 mmHg), crystalloid or colloid infusion, blood product transfusion, non-steroid anti-inflammatory drugs (NSAIDs) use, glucocorticoid medication, cumulative opioid consumption, and volatile anesthetic use.

### Propensity Score-Matched Analysis and Adjustment

Propensity score matching (PSM) was applied to address the potential residual confounding effect of the covariates such as demographic variables and clinical parameters, which would result in a biased outcome. The propensity score, a composite score, was computed based on the probability of patients having different levels of SII and derived from synthesized baseline parameters using the multivariate logistic regression model (Williamson and Forbes, 2014). After the propensity score generation, patients with SII < 583 and SII ≥ 583 were randomly matched at a 1:1 ratio using the greedy nearest-neighbor matching approach (maximum caliper width, 0.1). Kernel density plots of the propensity scores were applied to examine the equivalence between the matched patients. The standardized mean difference (SMD) was reported and performed to evaluate the baseline differences between the two groups, where a value < 0.1 indicated minor differences (Cheung et al., 2019).

### Statistical Analysis

Data were statistically analyzed using IBM SPSS Statistics for Windows, version 26.0 (IBM, Corp.) and R statistical software (R version 4.0.5, R Foundation for Statistical Computing). Continuous variables were summarized as mean [standard deviation (SD)], or median [interquartile range (IQR)]. Categorical data were displayed as numbers and percentages. The NLR or PLR score was converted into a two-level categorical variable based on the ROC analysis. The SII was also evaluated as continuous variable. We applied an extended multivariate logistic regression model approach to evaluate the clinical effect of the SII on perioperative ischemic stroke. Based on the type of surgical procedure, we conducted sensitivity analysis by only including non-neurosurgical patients to test the robustness of our findings. Subsequently, we further analyzed subgroups to appraise the primary correlation between the SII and perioperative ischemic stroke according to age, sex, intraoperative hypotension, diabetes mellitus, and prior ischemic stroke (Selim, 2007). The comparison group in this study referred to patients with SII < 583, unless stated otherwise. For all tests, statistical significance was inferred at a two-sided *P*-value < 0.05.

## Results

### Study Population

A total of 58,776 older patients who underwent non-cardiac surgery from January 2008 to August 2019 were enrolled in the study. Figure 1 illustrates the flow diagram of patient selection. After applying the inclusion and exclusion criteria, 40,670 eligible patients remained in the analysis, with a median age of 70 years (IQR: 67, 74), of whom 18,334 (45.1%) were women. Among the patients, 14,811 (36.4%) underwent intra-abdominal surgery, 1,903 (4.7%) underwent neurosurgery; and 6,224 (15.3%) received blood product transfusion. The prevalence of intraoperative hypotension (MAP ≤ 65 mmHg) in the cohort was 45.1% (*n* = 18,349). Of the entire patient cohort, 237 (0.58%) experienced an ischemic stroke within 30 days after surgery. The stroke incidence was consistent with previously reported rates of 0.1–0.7% in patients who underwent non-cardiac surgery (Vasivej et al., 2016; Woo et al., 2021).

The median NLR, PLR, and SII values were 2.00 (IQR:1.49, 2.83), 120.5 (IQR:93.1, 159.0), and 413.0 (IQR:287.0, 626.5), respectively. Using the ROC analysis, NLR, PLR, and SII accurately predicted perioperative ischemic stroke, with area under curve of 0.599 (95% CI: 0.534–0.632), 0.560 (95% CI: 0.505–0.616), and 0.721 (95% CI: 0.673–0.758), respectively. The Youden index of NLR, PLR, and SII were 0.165, 0.129, and 0.184, respectively. The optimal cut-off points derived for NLR, PLR, and SII to predict perioperative ischemic stroke were 3, 119, and 583 × 10^9^, respectively. The ROC curve of NLR, PLR, and SII for stroke risk classifier are shown in Supplementary Figures 1–3.

The patients were then grouped into low (< 583, *n* = 29,060, 71.5%) and high (≥ 583, *n* = 11,610, 28.5%) SII groups. The differences in baseline characteristics between the groups are presented in Table 1. Some baseline clinical characteristics in the study were relatively similar between the SII < 583 and SII ≥ 583 groups. However, other characteristics, such as sex, ASA classification, preoperative hemoglobin level, surgical procedures, duration of procedures, estimated blood loss, intraoperative hypotension, colloid infusion, blood transfusion, and opioid analgesic medication, differed between the two groups. Patients with SII ≥ 583 had more cardiovascular and cerebrovascular comorbidities (hypertension, diabetes mellitus, prior ischemic stroke, coronary heart disease, and peripheral vascular disease) and higher long-term administration of β-blockers and aspirin, than did those with SII < 583. Compared to patients with SII < 583, those with SII ≥ 583 had higher preoperative NLR and PLR scores.

**TABLE 1:** Baseline characteristics of unadjusted sample and propensity score-matched sample (patients from 2008–2019).

Characteristic	Unadjusted sample (*n* = 40,670)				PSM adjusted (1:1) (*n* = 21,652)			
	SII < 583 (*n* = 29,060)	SII ≥ 583 (*n* = 11,610)	*P-*value	SMD	SII < 583 (*n* = 10,826)	SII ≥ 583 (*n* = 10,826)	*P-*value	SMD
**Demographics**								
Age, y^†^	70.0 (67.0,73.0)	70.0 (67.0,75.0)	0.126	0.152	70.0 (67.0,74.0)	70.0 (67.0,74.0)	0.556	0.004
Female (%) ^†^	13651 (47.0)	4683 (40.3)	<0.001	0.134	4458 (41.2)	4427 (40.9)	0.679	0.006
BMI, kg/m^2†^	24.5 (22.3,26.9)	23.7 (21.5,26.0)	0.089	0.233	24.0 (21.6,26.4)	23.8 (21.5,26.0)	0.136	0.097
**ASA classification (%) ^†^**								
Class I	741 (2.5)	234 (2.0)	<0.001	0.197	261 (2.4)	230 (2.1)	0.356	0.022
Class II	22885 (78.8)	8255 (71.1)			7826 (72.3)	7793 (72.0)		
Class III	5434 (18.7)	3121 (26.9)			2739 (25.3)	2803 (25.9)		
**Previous medical history**								
Hypertension (%) ^†^	10874 (37.4)	4685 (40.4)	<0.001	0.060	4211 (38.9)	4360 (40.3)	0.257	0.021
Diabetes mellitus (%) ^†^	6096 (21.0)	2756 (23.7)	<0.001	0.066	2436 (22.5)	2554 (23.6)	0.178	0.076
Prior ischemic stroke (%) ^†^	1552 (5.3)	847 (7.3)	<0.001	0.080	682 (6.3)	765 (7.1)	0.228	0.068
Coronary heart disease (%) ^†^	2879 (9.9)	1231 (10.6)	0.037	0.023	1070 (9.9)	1146 (10.6)	0.093	0.023
Atrial fibrillation or VHD (%) ^†^	454 (1.6)	202 (1.7)	0.215	0.014	165 (1.5)	180 (1.7)	0.447	0.011
Peripheral vascular disease (%) ^†^	1996 (6.9)	892 (7.7)	0.004	0.031	811 (7.5)	802 (7.4)	0.836	0.003
Renal dysfunction (%) ^†^*	338 (1.2)	234 (2.0)	<0.001	0.068	191 (1.8)	205 (1.9)	0.456	0.047
β-blockers medication (%) ^†^	2051 (7.1)	999 (8.6)	<0.001	0.058	869 (8.2)	931 (8.6)	0.167	0.065
Aspirin medication (%) ^†^	2553 (8.8)	1174 (10.1)	<0.001	0.045	1024 (9.5)	1086 (10.0)	0.293	0.043
**Preoperative laboratory data**								
Hemoglobin, g/L^†^	132.0 (122.0,142.0)	125.0 (111.0,138.0)	<0.001	0.437	128.0 (114.0,140.0)	127.0 (113.0,139.0)	0.156	0.083
Albumin, g/L^†^	40.3 (38.1,42.5)	40.5 (38.2,43.0)	0.223	0.481	38.9 (36.2,41.4)	38.8 (36.0,41.7)	0.837	0.005
Total bilirubin, μmol/L^†^	10.9 (8.4,14.2)	10.6 (7.8,15.6)	<0.001	0.291	10.7 (8.3,14.6)	10.6 (7.7,14.9)	0.202	0.093
Prothrombin time, s^†^	13.1 (12.6,13.6)	13.2 (12.7,13.9)	0.123	0.176	13.2 (12.7,13.8)	13.2 (12.7,13.8)	0.600	0.028
**Surgical and anesthetic factors**								
Preoperative MAP, mmHg	95.7 (88.7,103.0)	95.0 (87.3,102.3)	0.098	0.070	95.0 (87.3,102.3)	95.0 (88.0,102.7)	0.169	0.024
**Surgical procedures (%)**								
Trauma surgery	433 (1.5)	602 (5.2)	<0.001	0.352	404 (3.7)	353 (3.3)		
Spine	2751 (9.5)	715 (6.2)			758 (7.0)	711 (6.6)	0.258	0.041
Intra-abdominal surgery	9652 (33.2)	5159 (44.4)			4688 (43.3)	4690 (43.3)		
Joint arthroplasty	3726 (12.8)	1031 (8.9)			987 (9.1)	1027 (9.5)		
Urologic or gynecologic	3972 (13.7)	1219 (10.5)			1138 (10.6)	1209 (11.1)		
Neurosurgery	1380 (4.7)	523 (4.5)			516 (4.8)	515 (4.8)		
Thoracic or vascular	3362 (11.6)	1225 (10.5)			1172 (10.8)	1199 (11.1)		
Other (plastic surgery, etc.)	3784 (13.0)	1136 (9.8)			1163 (10.7)	1122 (10.3)		
Duration of procedures, min	155.0 (110.0,215.0)	170.0 (120.0.0,235.0)	<0.001	0.162	168.0 (118.0,231.0)	170.0 (120.0,235.0)	0.356	0.076
Estimated blood loss, mL	100.0 (50.0,200.0)	150.0 (50.0,300.0)	<0.001	0.083	140.0 (90.0,280.0)	145.7 (100.0,300.0)	0.167	0.096
MAP ≤ 65 mmHg (%)	12600 (43.4)	5749 (49.5)	<0.001	0.070	5125 (47.3)	5285 (48.8)	0.234	0.072
Crystalloid infusion, ml/kg/h	8.6 (6.5,11.4)	8.9 (6.6,11.8)	0.167	0.073	8.8 (6.6,11.7)	8.8 (6.5,11.7)	0.845	0.006
Colloid infusion, ml/kg/h	2.9 (1.3,4.3)	3.1 (1.8,4.5)	<0.001	0.123	3.0 (1.6,4.4)	3.0 (1.8,4.5)	0.111	0.066
Blood transfusion (%)	3902 (13.4)	2322 (20.0)	<0.001	0.177	1998 (18.5)	2082 (19.2)	0.189	0.052
NSAIDs (%)	20502 (70.6)	8366 (72.1)	0.003	0.033	7667 (70.8)	7709 (71.2)	0.539	0.009
Glucocorticoid (%)	23749 (81.7)	9557 (82.3)	0.165	0.015	8905 (82.3)	8932 (82.5)	0.643	0.007
Opioid dose, mg^‡^	120.0 (9.0,150.0)	135.0 (105.0,165.0)	<0.001	0.081	135.0 (100.0,150.0)	135.0 (105.0,165.0)	0.256	0.047
Volatile anesthetic (%)	27098 (93.2)	10819 (93.2)	0.840	0.002	10097 (93.3)	10110 (93.4)	0.744	0.005
**Preoperative NLR**								
<3	27796 (95.7)	4098 (35.3)	<0.001	1.643	10215 (94.4)	3951 (36.5)	<0.001	1.583
≥3	1264 (4.3)	7512 (64.7)			611 (5.6)	6875 (63.5)		
**Preoperative PLR**								
<119	18897 (65.0)	959 (8.3)	<0.001	1.458	6821 (63.0)	914 (8.4)	<0.001	1.385
≥119	10163 (35.0)	10651 (91.7)			4005 (37.0)	9912 (91.6)		
Perioperative ischemic stroke (%)	126 (0.434)	111 (0.956)	<0.001	0.856	49 (0.453)	107 (0.988)	<0.001	0.939

*The data are presented as the median (inter-quartile range), mean (standard deviation) or n (%).*

*Creatinine > 177 μm/l.

*^†^Variables included in the propensity score.*

*^‡^Including those prescribed intraoperatively and postoperatively (until 7 days after surgery).*

*SII, systemic-immune-inflammation index; PSM, propensity score matching; SMD, standardized mean difference; BMI, body mass index; ASA, American Society of Anesthesiologists; VHD, valvular heart disease; MAP, mean arterial pressure; NSAIDs, non-steroid anti-inflammatory drugs; NLR, neutrophil-lymphocyte ratio; PLR, platelet-to-lymphocyte ratio.*

### Primary Analysis: Correlation Between SII and Perioperative Ischemic Stroke

We initially evaluated the association between the SII as continuous variable and perioperative ischemic stroke using univariate and multivariate logistic analyses. The univariate analysis showed that the SII was associated with perioperative ischemic stroke [odds ratio (OR): 1.285; 95% confidence interval (CI): 1.174–1.542; *P* < 0.001]. In the multivariate logistic regression model, the adjusted OR of SII was 1.213 (95% CI: 1.169–1.468; *P* < 0.001). The SII as continuous variable was an independent predictor of perioperative ischemic stroke (Supplementary Table 2).

When investigating the prognostic values of SII as categorical variable, the results of our univariate analysis revealed that age, hypertension, diabetes mellitus, prior ischemic stroke, coronary heart disease, arterial fibrillation, peripheral vascular disease, β-blockers use, aspirin medication, preoperative MAP, surgical procedures, duration of procedures, estimated blood loss, intraoperative hypotension (MAP ≤ 65 mmHg), crystalloid infusion, NSAIDs use, NLR, PLR, and SII were prognostic factors for perioperative ischemic stroke, whereas sex, BMI, ASA classification, renal dysfunction, preoperative laboratory data (hemoglobin, albumin, total bilirubin, and prothrombin time), colloid infusion, blood product transfusion, glucocorticoid use, opioid analgesic medication, and volatile anesthetic use had no prognostic significance (Supplementary Table 3). An elevated SII was strongly associated with an increased risk of perioperative ischemic stroke in the unadjusted analysis [OR: 2.217; 95% (CI): 1.714–2.863; *P* < 0.001] (Table 2).

**TABLE 2:** Association between SII and perioperative ischemic stroke using the logistic regression model and propensity score analysis.

Analysis method	OR	95% CI	*P-*value
**Logistic regression analysis (*n* = 40,670)**			
Model 1 (univariate model)*	2.217	1.714–2.863	<0.001
Model 2 (preoperative patient-related covariates adjusted)^†^	1.887	1.404–2.534	<0.001
Model 3 (Surgical and anesthetic covariates adjusted) ^‡^	2.301	1.768–2.989	<0.001
Model 4 (fully adjusted)^| |^	1.843	1.369–2.480	<0.001
**Propensity score analysis (multivariate)**			
Model PSM (*n* = 10,826)^¶^	2.195	1.574–3.106	<0.001

*SII, systemic-immune-inflammation index; OR, odds ratio; CI, confidence interval; PSM, propensity score matching.*

*Model 1 was a univariate regression model.

*^†^Model 2 included preoperative SII, age, female sex, BMI, ASA classification, hypertension, diabetes mellitus; prior ischemic stroke, coronary heart disease, arterial fibrillation, peripheral vascular disease, renal dysfunction, βblockers medication, aspirin medication, preoperative laboratory data (hemoglobin, albumin, total bilirubin, prothrombin time), preoperative NLR, preoperative PLR.*

*^‡^Model 3 included preoperative SII, preoperative MAP, surgical procedures, duration of procedures, estimated blood loss, MAP ≤ 65 mmHg, crystalloids infusion, colloids infusion, blood transfusion, NSAIDs, glucocorticoid, opioid dose, volatile anesthetic.*

*^| |^Model 4 included all the variables. Univariate and multivariate results are showed in Supplementary Table 3.*

*^¶^10,826 pairs were matched using propensity score method. Univariate and multivariate results are showed in Supplementary Table 4.*

Based on the extended multivariate logistic regression models (Table 2), patients with SII ≥ 583 showed a significantly higher OR of perioperative ischemic stroke in all three models consistently (OR range: 1.843–2.301, *P* < 0.001 for all) (Table 2). The NLR, but not PLR, was also an independent predictor of perioperative ischemic stroke in the adjusted analysis (OR: 1.036; 95% CI: 1.007–1.062; *P* = 0.007) (Supplementary Table 3). The other independent risk factors for predicting perioperative ischemic stroke are shown in Supplementary Table 3.

### Propensity Score-Matched Analysis and Adjustment

**FIGURE 2:**
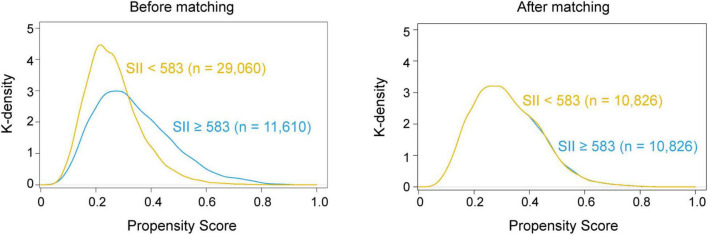
Distribution of propensity scores before and after matching.

Then, we performed PSM analysis to assess the prognostic value of the SII further. Prior to matching, the median propensity score in older patients with SII ≥ 583 was 0.266 (IQR: 0.203–0.377) vs. 0.231 (IQR: 0.194–0.346) in those without SII < 583. PSM resulted in 10,826 patients in the SII ≥ 583 group matched to 10,826 patients in the SII < 583 group. The distribution of propensity scores in the high and low SII groups is graphically illustrated before and after matching (Figure 2). After matching, the mean (SD) propensity score was similar between those with high [0.314 (0.127)] and low [0.318 (0.121)] SII. The baseline demographic and clinical characteristics of the variables were generally well balanced between the two groups, with SMD less than 0.10 for all covariates (Table 1). Following multivariate logistic regression adjustment after PSM (*n* = 10,826), the correlation between SII and perioperative ischemic stroke remained significantly robust (OR: 2.195; 95% CI: 1.574–3.106; *P* < 0.001) (Table 2 and Supplementary Table 4).

### Sensitivity Analysis

**FIGURE 3:**
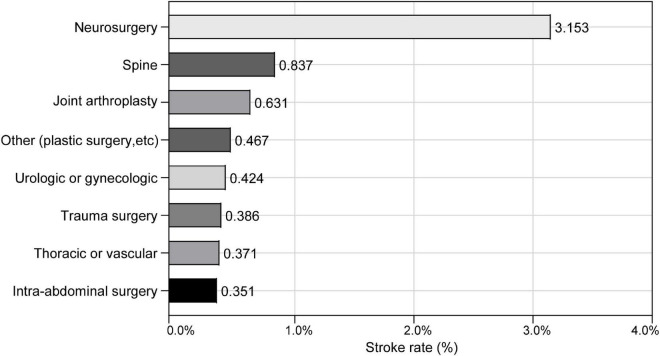
Incidence of perioperative ischemic stroke among the different surgical procedures.

The incidence of perioperative ischemic stroke is closely associated with the type and complexity of surgical procedures (Selim, 2007). The surgical procedures with the incidence of perioperative ischemic stroke at Chinese PLA General Hospital are shown in Figure 3. The surgical categories with highest overall incidence of perioperative ischemic stroke were neurosurgery (3.153%), followed by spine surgery (0.837%) and joint arthroplasty (0.631%). To further test the robustness of our results, we performed a sensitivity analysis excluding neurosurgeries. The adjusted OR of perioperative ischemic stroke in neurosurgical patients with the SII was 2.297 (95% CI: 1.192–4.429; *P* = 0.013). The association between preoperative SII and perioperative ischemic stroke remained stable in those non-neurosurgical patients (OR: 1.691; 95% CI: 1.202–2.376; *P* < 0.002) (Table 3).

**TABLE 3:** Sensitivity analysis of the association between SII and perioperative ischemic stroke (adjustment through multivariate logistic regression).

	SII < 583 and perioperative ischemic stroke cases, n	SII ≥ 583 and perioperative ischemic stroke cases, n	OR	95% CI	*P*-value
Entire cohort (*n* = 40,670; PIS = 237)	29,060 (126)	11,610 (111)	1.842	1.369–2.480	<0.001
**Type of surgery**					
Neurosurgery (*n* = 1,903; PIS = 60)	1,380 (28)	523 (32)	2.297	1.192–4.429	0.013
Non-neurosurgery (*n* = 38,767; PIS = 177)	27,680 (98)	11,087 (79)	1.691	1.202–2.376	0.002

*SII, systemic-immune-inflammation index; OR, odds ratio; CI, confidence interval; PIS, perioperative ischemic stroke.*

### Subgroup Analyses

**FIGURE 4:**
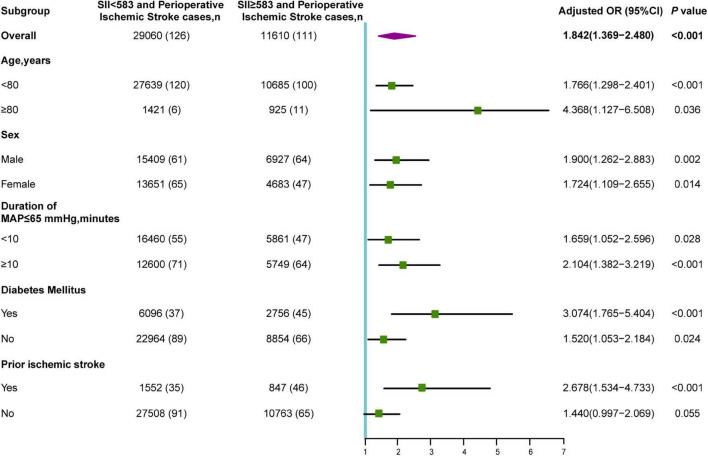
Subgroup analyses of the association between SII and perioperative ischemic stroke. OR, odds ratio; MAP, mean arterial pressure.

Among 11,610 older patients with SII ≥ 583, 925 (8.0%) were aged ≥ 80 years, 4,683 (40.3%) were female, 5,749 (49.5%) had a duration of intraoperative hypotension (MAP ≤ 65 mmHg) ≥ 10 min, 8,854 (23.7%) presented with diabetes mellitus, and 847 (7.3%) had prior ischemic stroke. The subgroup analyses for the prediction of a poor neurological outcome were summarized according to age, sex, intraoperative hypotension, diabetes mellitus, and prior ischemic stroke (Figure 4). The OR of the SII was significant for age subgroups [≥ 80 years: OR (95% CI): 4.368 (1.127–6.508), *P* = 0.036; < 80 years: OR (95% CI): 1.766 (1.298–2.401), *P* < 0.001]. Additionally, an increased risk of perioperative ischemic stroke was observed in both male (OR: 1.900; 95% CI: 1.262–2.883; *P* = 0.002) and female (OR: 1.724; 95% CI: 1.109–2.655; *P* = 0.014). In patients with suboptimal intraoperative hypotension (duration of MAP ≤ 65 mmHg ≥ 10 min), the SII was significantly correlated with perioperative ischemic stroke (OR: 2.104; 95% CI: 1.382–2.184; *P* < 0.001). This increased risk was also significant in those with intraoperative hypotension (MAP duration ≤ 65 mmHg < 10 min). The correlation between preoperative SII and perioperative ischemic stroke was significant in individuals with (OR: 3.074; 95% CI: 1.765–5.404; *P* < 0.001) and without (OR: 1.520; 95% CI: 1.053–2.184; *P* = 0.024) diabetes. Additionally, an elevated SII was only significantly associated with an increased risk of perioperative ischemic stroke in the prior ischemic stroke group (OR: 2.678; 95% CI: 1.534–4.733; *P* < 0.001), whereas it was not significant in the no prior ischemic stroke group (OR: 1.440; 95% CI: 0.997–2.069; *P* = 0.055).

## Discussion

Using this cohort of 40,670 older elderly patients who had undergone non-cardiac surgery, we evaluated the prognostic value of inflammatory biomarkers or indices in predicting perioperative ischemic stroke. Our analysis indicated that preoperative elevated SII was substantially correlated with an increased risk of perioperative ischemic stroke. In addition, after adjustment, NLR, but not PLR, was an independent prognostic factor for perioperative ischemic stroke.

Extensive evidence has shown that the disruption of immune state homeostasis and inflammation are closely interrelated with the occurrence and progression of various diseases. In particular, immune checkpoint inhibitors have been approved to treat several cancers (Johansson et al., 2016). As such, the potential predictive values of inflammation-related biomarkers or inflammation-based scores have been exploited to improve diagnostic precision and predict disease progression. Given their easy accessibility, appropriate cost-effectiveness, and universal applicability, the NLR, PLR, and SII deserve more attention. Previous studies have indicated that the NLR is a significant independent risk factor for tumorigenesis, tumor invasion, and metastasis (Mantovani et al., 2008; Gukovsky et al., 2013). In addition, PLR was significantly correlated with adverse postoperative outcomes, including death, and cardiovascular and cerebrovascular events in cardiac or non-cardiac surgeries (De Giorgi et al., 2019; Dey et al., 2021). The SII was developed based on a combination of inflammatory cells (neutrophils and lymphocytes) and thrombotic factor (platelets). A high SII independently predicted recurrence and mortality in patients with pancreatic cancer or gastroesophageal adenocarcinoma (Aziz et al., 2019; Jomrich et al., 2021). Additionally, the association of elevated SII (> 694.3 × 10^9^) with postoperative major adverse cardiovascular events was elucidated in individuals with coronary heart disease (Yang et al., 2020). An increased SII (≥ 455.6 × 10^9^) also showed significant prognostic value for predicting all-cause mortality and major postoperative complications in patients undergoing isolated tricuspid valve surgery (Yoon et al., 2021).

Utilizing biomarkers to stratify high-risk patients and predict perioperative ischemic stroke remains an elusive and major challenge in clinical decision-making. For instance, in this study, PLR did not exhibit significant predictive values in older patients undergoing non-cardiac surgery, whereas NLR appeared to be a relatively weak risk factor. Consistent with prior studies, the predictive values of NLR and PLR remained inconsistent and controversial (Li et al., 2020; Yoon et al., 2021). However, we demonstrated that the preoperative elevated SII exhibited a favorable prognostic value for perioperative ischemic stroke in the univariate and extended multivariate logistic regression models. The SII maintained a good prognostic value in neurosurgical and non-neurosurgical patients, indicating the reliability of the prognostic ability in different surgical populations. Going further, we revealed that preoperative increased SII was a potential risk indicator for increased risk of perioperative ischemic stroke in non-cardiac surgery patients, particularly in patients ≥ 80 years of age, male, with intraoperative hypotension, with diabetes, and with prior ischemic stroke. These results implied that the preoperative SII might potentially emerge as a simple, promising, and useful prognostic biomarker for perioperative ischemic stroke prediction in older patients who underwent non-cardiac surgery. In particular, preoperative SII showed broader applicability as a useful prognostic indicator due to the easy accessibility and appropriate cost-effectiveness.

Several possible explanations may account for the association between preoperative SII and perioperative ischemic stroke. Elevated SII was most often observed in patients with neutrophilia, lymphopenia and thrombocythemia, indicating an integration of compromised innate responses and aberrant adaptive immune responses. Moreover, immune-mediated brain damage has gained increasing attention and recognition. Accumulating evidence indicates a critical role of neuroinflammation in the pathogenesis of perioperative ischemic stroke and secondary brain injury.

The aggregation and migration of neutrophils are closely involved in ischemic brain damage, disruption of blood-brain barrier integrity, and functional recovery after stroke (Shi et al., 2020). Activated neutrophils can also damage vascular endothelial cells and brain tissues by releasing toxic reactive oxygen species and proteases such as matrix metalloproteinase-9, resulting in acute neuroinflammation (Prasad et al., 2011). Thrombocytosis is correlated with inflammatory conditions in patients with thromboembolism (Boyle et al., 2013). Initial platelet plug formation, agglutination and platelet thromboinflammatory responses are important for blood clotting, systemic inflammatory reactions, and endothelial reorganization (Sprague et al., 2008; Chan et al., 2015; Telen, 2016). In contrast, lymphopenia may lead to the temporary suspension of some mechanisms, which normally maintain the balance between host tolerance and host defense (Baker et al., 2017). In addition, neutrophil-platelet interactions play a role in modulating the function of these cells and precipitating vascular inflammation (von Brühl et al., 2012; Kim et al., 2015). Furthermore, perioperative factors, including surgery and general anesthesia, may amplify the risk of preoperative aberrant innate and adaptive immune states, particularly in older surgical patients. In this context, in our study, the preoperative SII may be even more promising as a predictive biomarker than NLR and PLR, due to the combined effect of neutrophil, lymphocyte, and platelet counts.

This cohort study has several strengths. First, to our knowledge, no previous studies have assessed the prognostic value of preoperative SII in older patients undergoing non-cardiac surgery. Second, considering the low incidence of perioperative ischemic stroke, we attempted to incorporate as many patients as possible and finally included 40,670 eligible patients in our study. Third, we used the PSM analysis adjusted for several potentially confounding variables, including patient demographics, preoperative medical history, and preoperative laboratory data. Fourth, multiple statistical approaches, such as sensitivity and subgroup analyses, were successfully performed to confirm that the preoperative SII qualifies as a promising, independent prognostic indicator of perioperative ischemic stroke in older patients undergoing non-cardiac surgery.

This study has several inevitable limitations that warrant acknowledgement. First, owing to its observational nature, we could only demonstrate associations but not causality. Thus, prospective and properly designed studies are urgently needed to confirm and validate the cause-and-effect relationship between preoperative SII and perioperative ischemic stroke in older patients. Second, our study is a single-center design, which limits its generalizability. Thus, our findings are hypothesis-generating, and further external validation of our results is important before generalizing the SII in clinical use. Third, since high-sensitivity CRP and procalcitonin are not routinely tested in clinical settings, we did not include these two inflammatory biomarkers, which may have introduced bias. Fourth, although we carefully adjusted for a multitude of potential confounders, residual confounding and unmeasured factors cannot be entirely ruled out.

## Conclusion

In conclusion, the preoperative SII was an independent prognostic factor in older patients undergoing non-cardiac surgery. The SII components are routinely collected in the clinical settings, thus the SII qualifies as a novel, promising prognostic index after corroboration by subsequent larger studies. Our findings highlight the clinical importance of inflammation-based markers and the essential role of the immune system in perioperative ischemic stroke. Nevertheless, the SII requires more properly designed studies to validate its prognostic value in older patients undergoing non-cardiac surgery.

## Data Availability Statement

The original contributions presented in the study are included in the article/Supplementary Material, further inquiries can be directed to the corresponding author/s.

## Ethics Statement

The studies involving human participants were reviewed and approved by the Medical Ethics Committee of Chinese PLA General Hospital. The ethics committee waived the requirement of written informed consent for participation.

## Author Contributions

JL and YM: conception and design. FZ, MN, and LW: development of methodology. FZ and JC: acquisition of data. MN and LS: analysis and interpretation of data. WM and YL: supervise the study. FZ, MN, LW, JL, and YM: drafting, review, and revision of the manuscript. All authors approved the final manuscript.

## Conflict of Interest

The authors declare that the research was conducted in the absence of any commercial or financial relationships that could be construed as a potential conflict of interest.

## Publisher’s Note

All claims expressed in this article are solely those of the authors and do not necessarily represent those of their affiliated organizations, or those of the publisher, the editors and the reviewers. Any product that may be evaluated in this article, or claim that may be made by its manufacturer, is not guaranteed or endorsed by the publisher.
