# Corrigendum: Systemic-immune-inflammation index as a promising biomarker for predicting perioperative ischemic stroke in older patients who underwent non-cardiac surgery

**DOI:** 10.3389/fnagi.2022.1101574

**Published:** 2022-12-08

**Authors:** Faqiang Zhang, Mu Niu, Long Wang, Yanhong Liu, Likai Shi, Jiangbei Cao, Weidong Mi, Yulong Ma, Jing Liu

**Affiliations:** ^1^Anesthesia and Operation Center, The First Medical Center, Chinese PLA General Hospital, Beijing, China; ^2^Department of Neurology, The Affiliated Hospital of Xuzhou Medical University, Xuzhou Medical University, Xuzhou, China; ^3^Department of Pain Medicine, The First Medical Center, Chinese PLA General Hospital, Beijing, China

**Keywords:** systemic-immune-inflammation index (SII), perioperative stroke, postoperative complication, inflammation, older patients, biomarker

In the original article, there was a mistake in Figure 1 as published. “ASA physical status V” should be “ASA physical status ≥ IV.” In addition the corresponding *n* number was give as 391, but should be 1,091. In addition, the corresponding n number for “Missing data for any confounder” was 3,144 but should be 2,444. The revised Figure 1 appears below.

**Figure 1:**
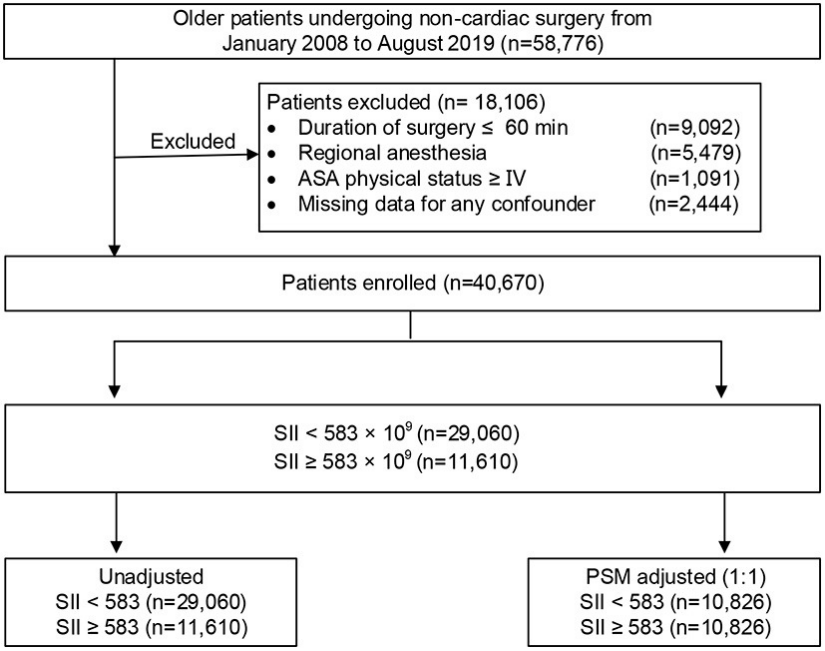
Study profile. ASA, American Society of Anesthesiologists; PSM, propensity score matching.

There was also an error in **Materials and methods**, “*Inclusion and exclusion criteria*,” Paragraph 1. “Patients who presented with an American Society of Anesthesiologists (ASA) classification of V” should be “Patients who presented with an American Society of Anesthesiologists (ASA) classification of ≥IV.” The corrected paragraph appears below:

“Patients who underwent non-cardiac surgery between January 2008 and August 2019 at Chinese PLA General Hospital were initially screened from a perioperative retrospective database. The inclusion criteria were as follows: (1) aged 65 yr or older; (2) underwent non-cardiac surgery; (3) received general anesthesia; and (4) were with duration of surgery > 60 min. Patients who presented with an American Society of Anesthesiologists (ASA) classification of ≥IV, were performed under regional anesthesia, or had missing clinical data were excluded. Among patients who underwent multiple surgeries during the study period, only the first eligible surgery was considered. A flow diagram of the patient selection process is displayed in Figure 1.” In the original article, there was also a mistake in the **Abstract**, “*Conclusion*” as published. The Abstract conclusion stated, “after non-cardiac surgery in elderly older patients.” This should be “after non-cardiac surgery in older patients.” The corrected paragraph appears below:

“**Conclusion:** Preoperative SII, which includes neutrophil, platelet, and lymphocyte counts obtained from routine blood analysis, was a potential prognostic biomarker for predicting perioperative ischemic stroke after non-cardiac surgery in older patients. An elevated SII, based on an optimal cut-off value of 583, was an independent risk factor for perioperative ischemic stroke.”

In the original article, there was also an error in Table 1 and Supplementary Tables 2–4. The covariates previously stated “Class III and IV” and “Arterial fibrillation.” The corrected covariates are “Class III” and “Atrial fibrillation or VHD.” The corrected Table 1 appears below. The corrected Supplementary Tables 2–4 appear in the Supplementary Material of the original article.

**Table 1:** Baseline characteristics of unadjusted sample and propensity score-matched sample (patients from 2008–2019).

**Characteristic**	**Unadjusted sample** **(*****n*** = **40,670)**				**PSM adjusted (1:1)** **(*****n*** = **21,652)**			
	**SII < 583**	**SII ≥583**	***P-*value**	**SMD**	**SII < 583**	**SII ≥583**	***P-*value**	**SMD**
	**(*n* = 29,060)**	**(*n* = 11,610)**			**(*n* = 10,826)**	**(*n* = 10,826)**		
**Demographics**								
Age, y^†^	70.0 (67.0,73.0)	70.0 (67.0,75.0)	0.126	0.152	70.0 (67.0,74.0)	70.0 (67.0,74.0)	0.556	0.004
Female (%)^†^	13651 (47.0)	4683 (40.3)	< 0.001	0.134	4458 (41.2)	4427 (40.9)	0.679	0.006
BMI, kg/m^2^^†^	24.5 (22.3,26.9)	23.7 (21.5,26.0)	0.089	0.233	24.0 (21.6,26.4)	23.8 (21.5,26.0)	0.136	0.097
**ASA classification (%)** ^†^								
Class I	741 (2.5)	234 (2.0)	< 0.001	0.197	261 (2.4)	230 (2.1)	0.356	0.022
Class II	22885 (78.8)	8255 (71.1)			7826 (72.3)	7793 (72.0)		
Class III	5434 (18.7)	3121 (26.9)			2739 (25.3)	2803 (25.9)		
**Previous medical history**								
Hypertension (%)^†^	10874 (37.4)	4685 (40.4)	< 0.001	0.060	4211 (38.9)	4360 (40.3)	0.257	0.021
Diabetes mellitus (%)^†^	6096 (21.0)	2756 (23.7)	< 0.001	0.066	2436 (22.5)	2554 (23.6)	0.178	0.076
Prior ischemic stroke (%)^†^	1552 (5.3)	847 (7.3)	< 0.001	0.080	682 (6.3)	765 (7.1)	0.228	0.068
Coronary heart disease (%)^†^	2879 (9.9)	1231 (10.6)	0.037	0.023	1070 (9.9)	1146(10.6)	0.093	0.023
Atrial fibrillation or VHD (%)^†^	454 (1.6)	202 (1.7)	0.215	0.014	165 (1.5)	180 (1.7)	0.447	0.011
Peripheral vascular disease (%)^†^	1996 (6.9)	892 (7.7)	0.004	0.031	811 (7.5)	802 (7.4)	0.836	0.003
Renal dysfunction (%)^†^^*^	338 (1.2)	234 (2.0)	< 0.001	0.068	191 (1.8)	205 (1.9)	0.456	0.047
β-blockers medication (%)^†^	2051 (7.1)	999 (8.6)	< 0.001	0.058	869 (8.2)	931 (8.6)	0.167	0.065
Aspirin medication (%)^†^	2553 (8.8)	1174 (10.1)	< 0.001	0.045	1024 (9.5)	1086 (10.0)	0.293	0.043
**Preoperative laboratory data**								
Hemoglobin, g/L^†^	132.0 (122.0,142.0)	125.0 (111.0,138.0)	< 0.001	0.437	128.0 (114.0,140.0)	127.0 (113.0,139.0)	0.156	0.083
Albumin, g/L^†^	40.3 (38.1,42.5)	40.5 (38.2,43.0)	0.223	0.481	38.9 (36.2,41.4)	38.8 (36.0,41.7)	0.837	0.005
Total bilirubin, μmol/L^†^	10.9 (8.4,14.2)	10.6 (7.8,15.6)	< 0.001	0.291	10.7 (8.3,14.6)	10.6 (7.7,14.9)	0.202	0.093
Prothrombin time, s^†^	13.1 (12.6,13.6)	13.2 (12.7,13.9)	0.123	0.176	13.2 (12.7,13.8)	13.2 (12.7,13.8)	0.600	0.028
**Surgical and anesthetic factors**								
Preoperative MAP, mmHg	95.7 (88.7,103.0)	95.0 (87.3,102.3)	0.098	0.070	95.0 (87.3,102.3)	95.0 (88.0,102.7)	0.169	0.024
**Surgical procedures (%)**								
Trauma surgery	433 (1.5)	602 (5.2)	< 0.001	0.352	404 (3.7)	353 (3.3)		
Spine	2751 (9.5)	715 (6.2)			758 (7.0)	711 (6.6)	0.258	0.041
Intra-abdominal surgery	9652 (33.2)	5159 (44.4)			4688 (43.3)	4690 (43.3)		
Joint arthroplasty	3726 (12.8)	1031 (8.9)			987 (9.1)	1027 (9.5)		
Urologic or gynecologic	3972 (13.7)	1219 (10.5)			1138 (10.6)	1209 (11.1)		
Neurosurgery	1380 (4.7)	523 (4.5)			516 (4.8)	515 (4.8)		
Thoracic or vascular	3362 (11.6)	1225 (10.5)			1172 (10.8)	1199 (11.1)		
Other (plastic surgery, etc.)	3784 (13.0)	1136 (9.8)			1163 (10.7)	1122 (10.3)		
Duration of procedures, min	155.0 (110.0,215.0)	170.0 (120.0.0,235.0)	< 0.001	0.162	168.0 (118.0,231.0)	170.0 (120.0,235.0)	0.356	0.076
Estimated blood loss, mL	100.0 (50.0,200.0)	150.0 (50.0,300.0)	< 0.001	0.083	140.0 (90.0,280.0)	145.7 (100.0,300.0)	0.167	0.096
MAP ≤ 65 mmHg (%)	12600 (43.4)	5749 (49.5)	< 0.001	0.070	5125 (47.3)	5285 (48.8)	0.234	0.072
Crystalloid infusion, ml/kg/h	8.6 (6.5,11.4)	8.9 (6.6,11.8)	0.167	0.073	8.8 (6.6,11.7)	8.8 (6.5,11.7)	0.845	0.006
Colloid infusion, ml/kg/h	2.9 (1.3,4.3)	3.1 (1.8,4.5)	< 0.001	0.123	3.0 (1.6,4.4)	3.0 (1.8,4.5)	0.111	0.066
Blood transfusion (%)	3902 (13.4)	2322 (20.0)	< 0.001	0.177	1998 (18.5)	2082 (19.2)	0.189	0.052
NSAIDs (%)	20502 (70.6)	8366 (72.1)	0.003	0.033	7667 (70.8)	7709 (71.2)	0.539	0.009
Glucocorticoid (%)	23749 (81.7)	9557 (82.3)	0.165	0.015	8905 (82.3)	8932 (82.5)	0.643	0.007
Opioid dose, mg^‡^	120.0 (9.0,150.0)	135.0 (105.0,165.0)	< 0.001	0.081	135.0 (100.0,150.0)	135.0 (105.0,165.0)	0.256	0.047
Volatile anesthetic (%)	27098 (93.2)	10819 (93.2)	0.840	0.002	10097 (93.3)	10110 (93.4)	0.744	0.005
**Preoperative NLR**								
< 3	27796 (95.7)	4098 (35.3)	< 0.001	1.643	10215 (94.4)	3951 (36.5)	< 0.001	1.583
≥3	1264 (4.3)	7512 (64.7)			611 (5.6)	6875 (63.5)		
**Preoperative PLR**								
< 119	18897 (65.0)	959 (8.3)	< 0.001	1.458	6821 (63.0)	914 (8.4)	< 0.001	1.385
≥119	10163 (35.0)	10651 (91.7)			4005 (37.0)	9912 (91.6)		
Perioperative ischemic stroke (%)	126 (0.434)	111 (0.956)	< 0.001	0.856	49 (0.453)	107 (0.988)	< 0.001	0.939

The data are presented as the median (inter-quartile range), mean (standard deviation) or n (%).

* Creatinine > 177 μm/l.

† Variables included in the propensity score.

‡ Including those prescribed intraoperatively and postoperatively (until 7 days after surgery).

SII, systemic-immune-inflammation index; PSM, propensity score matching; SMD, standardized mean difference; BMI, body mass index; ASA, American Society of Anesthesiologists; VHD, valvular heart disease; MAP, mean arterial pressure; NSAIDs, non-steroid anti-inflammatory drugs; NLR, neutrophil-lymphocyte ratio; PLR, platelet-to-lymphocyte ratio.

In the original article there was also an error in **Materials and methods**, “*Clinical outcome*.” The definition of perioperative ischemic stroke was incomplete. The following information was not provided: “Diagnoses of stroke are confirmed by a combination of neuroimaging and clinical evidence of cerebrovascular ischemia during hospital stay.” The corrected paragraph appears below:

“The primary outcome of interest was perioperative ischemic stroke, defined as an episode of neurological dysfunction, such as motor, sensory, or cognitive dysfunction, caused by focal cerebral, spinal, or retinal infarction within 30 postoperative days (Sacco et al., 2013). Diagnoses of stroke are confirmed by a combination of neuroimaging and clinical evidence of cerebrovascular ischemia during hospital stay. In our study, perioperative ischemic stroke patients were identified if discharge records included at least 1 ICD-9-CM/ICD-10-CM diagnosis code for stroke (Supplementary Table 1).”

We apologize for these errors and state that this does not change the scientific conclusions of the article in any way. The original article has been updated.
