# Supplementary material for: Systemic-Immune-Inflammation Index as a Promising Biomarker for Predicting Perioperative Ischemic Stroke in Older Patients Who Underwent Non-cardiac Surgery
Source: Front Aging Neurosci. 2022 Apr 1;14:865244. doi: 10.3389/fnagi.2022.865244 (PMC9010030; doi:10.3389/fnagi.2022.865244)
Supplement: Supplementary file 1 [file Table_1.doc]

SUPPLEMENTAL MATERIAL

**Supplementary Table 1**. Comorbidity and outcome definitions based on International Classification of Diseases, Ninth/Tenth Revision (ICD-9/10) codes.

| Comorbidity | Code type | Code | Description |
| --- | --- | --- | --- |
| Arterial  hypertension | ICD-9/ICD-10 | 401.X/I10.X | Essential hypertension |
| Diabetes  mellitus with  and without  chronic  complications | ICD-10 | E10.0X | Type 1 DM |
|  | ICD-10 | E10.1X | Type 1 DM with ketoacidosis |
|  | ICD-10 | E10.6X | Type 1 DM with other specified complications |
|  | ICD-10 | E10.8X | Type 1 DM with unspecified complications |
|  | ICD-10 | E10.9X | Type 1 DM without complications |
|  | ICD-10 | E11.0X | Type 2 DM with hyperosmolarity |
|  | ICD-10 | E11.1X | Type 2 DM with hyperosmolarity with coma |
|  | ICD-10 | E11.6X | Type 2 DM with other specified complications |
|  | ICD-10 | E11.8X | Type 2 DM with unspecified complications |
|  | ICD-10 | E11.9X | Type 2 DM without complications |
|  | ICD-10 | E12.0X | DM associated with malnutrition |
|  | ICD-10 | E12.1X | DM associated with malnutrition with coma |
|  | ICD-10 | E12.6X | DM associated with malnutrition with other specified  complications |
|  | ICD-10 | E12.8X | DM associated with malnutrition with unspecified complications |
|  | ICD-10 | E12.9X | DM associated with malnutrition without complications |
|  | ICD-10 | E13.0X | Other specified DM with hyperosmolarity |
|  | ICD-10 | E13.1X | Other specified DM with ketoacidosis |
|  | ICD-10 | E13.6X | Other specified DM with other specified complications |
|  | ICD-10 | E13.8X | Other specified DM with unspecified complications |
|  | ICD-10 | E13.9X | Other specified DM without complications |
|  | ICD-10 | E14.0X | DM, not elsewhere classified, with coma |
|  | ICD-10 | E14.1X | DM, not elsewhere classified, with ketoacidosis |
|  | ICD-9/ICD-10 | 250.8/E14.6X | Unspecified DM with other specified complications |
|  | ICD-9/ICD-10 | 250.9/E14.8X | Unspecified DM with other specified complications |
|  | ICD-10 | E14.9X | DM, not elsewhere classified, without complications |
|  | ICD-9 | 250.1 | DM with ketoacidosis |
|  | ICD-9 | 250.0 | DM without complications |
|  | ICD-9 | 250.2 | DM with hyperosmolarity |
|  | ICD-9 | 250.3 | DM with other coma |
|  | ICD-10 | E10.2X | Type 1 DM with kidney complications |
|  | ICD-10 | E10.3X | Type 1 DM with ophthalmic complications |
|  | ICD-10 | E10.4X | Type 1 DM with neurological complications |
|  | ICD-10 | E10.5X | Type 1 DM with circulatory complications |
|  | ICD-10 | E10.7X | Type 1 DM with multiple complications |
|  | ICD-10 | E11.2X | Type 2 DM with kidney complications |
|  | ICD-10 | E11.3X | Type 2 DM with ophthalmic complications |
|  | ICD-10 | E11.4X | Type 2 DM with neurological complications |
|  | ICD-10 | E11.5X | Type 2 DM with circulatory complications |
|  | ICD-10 | E11.7X | Type 2 DM with multiple complications |
|  | ICD-10 | E12.2X | DM associated with malnutrition with renal complications |
|  | ICD-10 | E12.3X | DM associated with malnutrition with ophthalmic complications |
|  | ICD-10 | E12.4X | DM associated with malnutrition with neurological complications |
|  | ICD-10 | E12.5X | DM associated with malnutrition with peripheral vascular complications |
|  | ICD-10 | E12.7X | DM associated with malnutrition with multiple complications |
|  | ICD-10 | E13.2X | Other specified DM with kidney complications |
|  | ICD-10 | E13.3X | Other specified DM with ophthalmic complications |
|  | ICD-10 | E13.4X | Other specified DM with neurological complications |
|  | ICD-10 | E13.5X | Other specified DM with circulatory complications |
|  | ICD-10 | E13.7X | Other specified DM with multiple complications |
|  | ICD-10 | E14.2X | DM, not elsewhere specified, with renal complications |
|  | ICD-10 | E14.3X | DM, not elsewhere specified, with ophthalmic complications |
|  | ICD-10 | E14.4X | DM, not elsewhere specified, with neurological complications |
|  | ICD-10 | E14.5X | DM, not elsewhere specified, with peripheral vascular  complications |
|  | ICD-10 | E14.7X | DM, not elsewhere specified, with multiple complications |
|  | ICD-9 | 250.4 | Diabetes with renal complications |
|  | ICD-9 | 250.5 | Diabetes with ophthalmic complications |
|  | ICD-9 | 250.6 | Diabetes with neurological complications |
|  | ICD-9 | 250.7 | Diabetes with peripheral circulatory disorders |
| Coronary heart disease | ICD-10 | I24.01 | Acute coronary thrombosis not resulting in myocardial infarction |
|  | ICD-10 | I24.81 | Other forms of acute ischemic heart disease |
|  | ICD-10 | I24.91 | Acute ischemic heart disease, unspecified |
|  | ICD-10 | I25.0 | Chronic ischemic heart disease |
|  | ICD-10 | I25.10 | Atherosclerotic heart disease of native coronary artery |
|  | ICD-10 | I25.101 | Atherosclerotic heart disease of native coronary artery without angina pectoris |
|  | ICD-10 | I25.110 | Atherosclerotic heart disease of native coronary artery with angina pectoris |
|  | ICD-10 | I25.1101 | Atherosclerotic heart disease of native coronary artery with unstable angina pectoris |
|  | ICD-10 | I25.1111 | Atherosclerotic heart disease of native coronary artery with angina pectoris with documented spasm |
|  | ICD-10 | I25.1118 | Atherosclerotic heart disease of native coronary artery with other forms of angina pectoris |
|  | ICD-10 | I25.1119 | Atherosclerotic heart disease of native coronary artery with unspecified angina pectoris |
|  | ICD-10 | I25.411 | Coronary artery aneurysm |
|  | ICD-10 | I25.421 | Coronary artery dissection |
| Atrial fibrillation | ICD-9/ICD-10 | 427.3X/I48.X | Atrial fibrillation and flutter |
| Peripheral  vascular  disease | ICD-9/ICD-10 | 440.X/I70.X | Atherosclerosis |
|  | ICD-9/ICD-10 | 441.X/I71.X | Aortic aneurysm and dissection |
|  | ICD-9/ICD-10 | 443.1/I73.1 | Thromboangiitis obliterans (Buerger's disease) |
|  | ICD-9/ICD-10 | 443.8X/I73.8X | Other specified peripheral vascular diseases |
|  | ICD-9/ICD-10 | 443.9/I73.9 | Peripheral vascular disease, unspecified |
|  | ICD-9/ICD-10 | 447.1/I77.1 | Stricture of artery |
|  | ICD-10 | I79.0X | Aneurysm of aorta in diseases classified elsewhere |
|  | ICD-10 | I79.2X | Peripheral angiopathy |
|  | ICD-9/ICD-10 | 557.1/K55.1X | Chronic vascular disorders of intestine |
|  | ICD-10 | K55.8X | Other vascular disorders of intestine |
|  | ICD-9/ICD-10 | 557.9/K55.9X | Vascular disorders of intestine, unspecified |
|  | ICD-9/ICD-10 | V43.4/Z95.8X | Presence of other cardiac and vascular implants and grafts |
|  | ICD-10 | Z95.9X | Presence of cardiac and vascular implant and graft, unspecified |
|  | ICD-9 | 442.X | Other aneurysm |
|  | ICD-9 | 443.2X | Other arterial dissection |
|  | ICD-9/ICD-10 | 440.2, I70.2 | Peripheral arterial disease |
|  | ICD-9/ICD-10 | 440.3, I70.3,  I70.5, I70.6,  I70.7 | Atherosclerosis of bypass graft of the extremities |
|  | ICD-9/ICD-10 | 440.4, I70.92 | Chronic total occlusion of artery of the extremities |
|  | ICD-9 | 443.9 | Peripheral vascular disease, unspecified |
| Ischemic stroke | ICD-9/ICD-10 | 433.X1/I63.X | Occlusion and stenosis of precerebral arteries with cerebral  infarction |
|  | ICD-9 | 434.X1 | Occlusion of cerebral arteries with cerebral infarction |
|  | ICD-9/ICD-10 | 437.1/I67.81,  I67.89 | Other generalized ischemic cerebrovascular disease |
|  | ICD-9/ICD-10 | 437.9/I67.9 | Unspecified cerebrovascular disease |

**
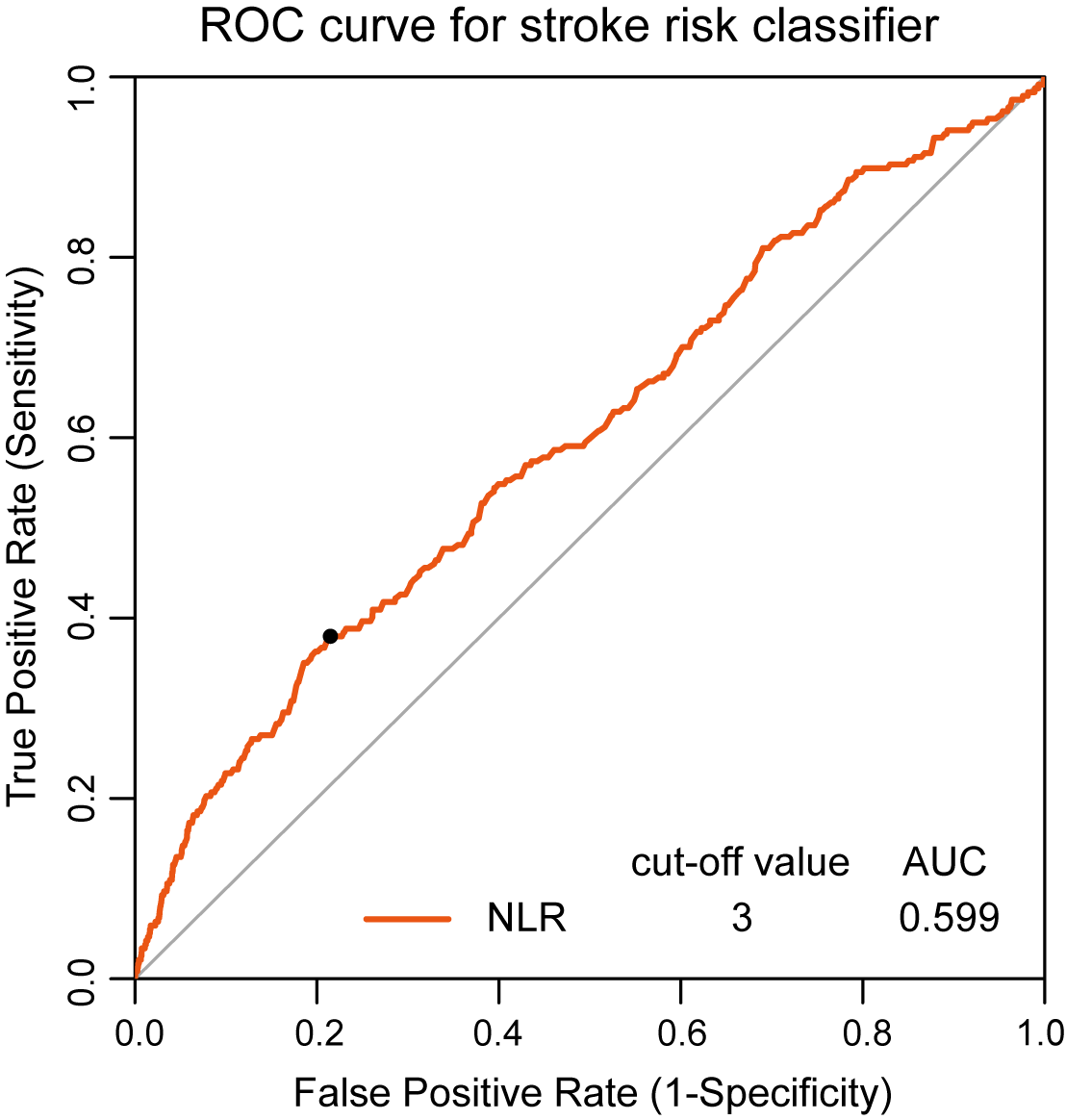
**

**Supplementary Figure 1**. ROC curve of NLR for perioperative ischemic stroke. ROC, receiver operating characteristics curve; NLR, neutrophil-lymphocyte ratio; AUC, area under curve.

**
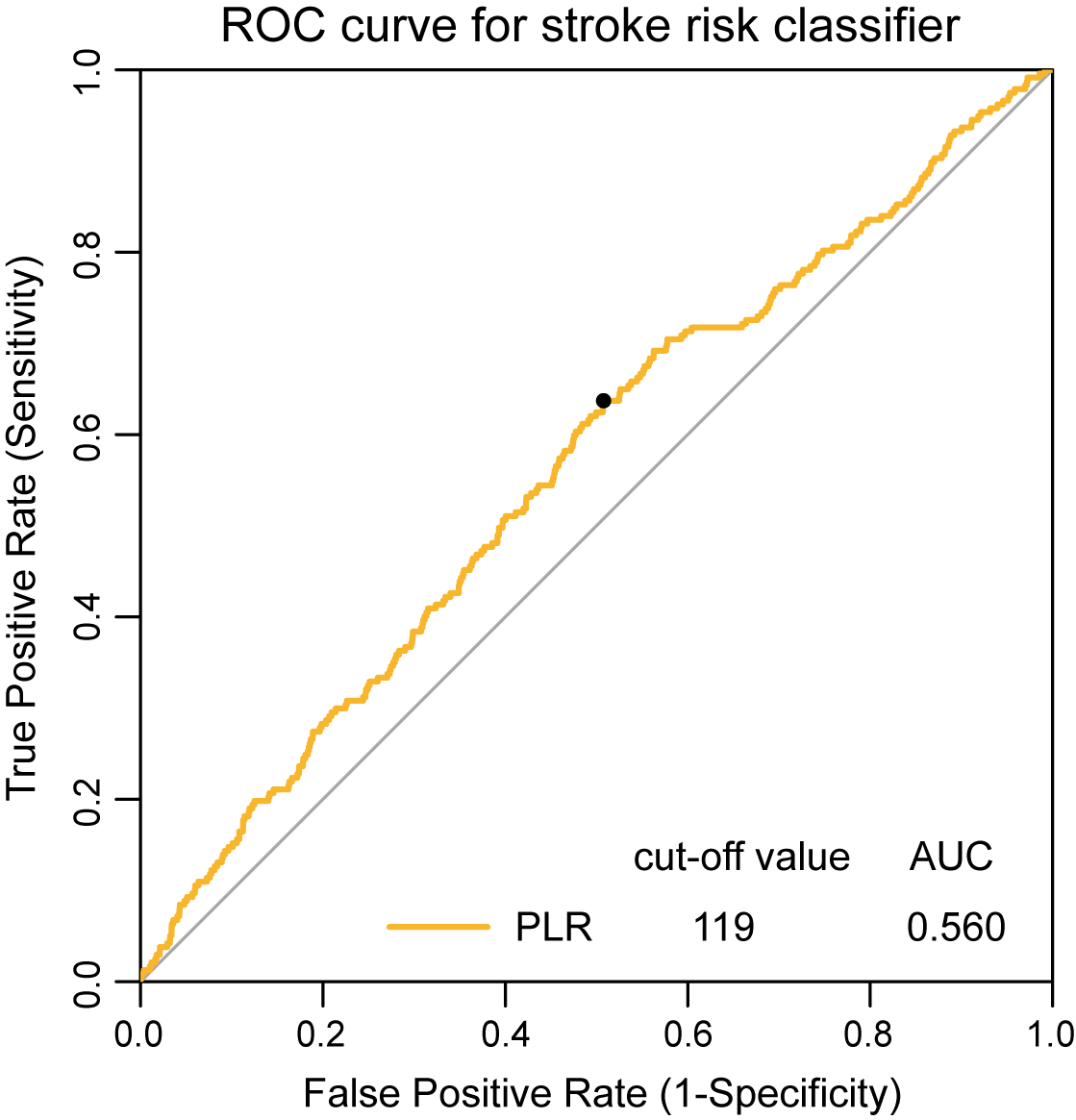
**

**Supplementary Figure 2**. ROC curve of PLR for perioperative ischemic stroke. ROC, receiver operating characteristics curve; PLR, platelet-to-lymphocyte ratio; AUC, area under curve.

**
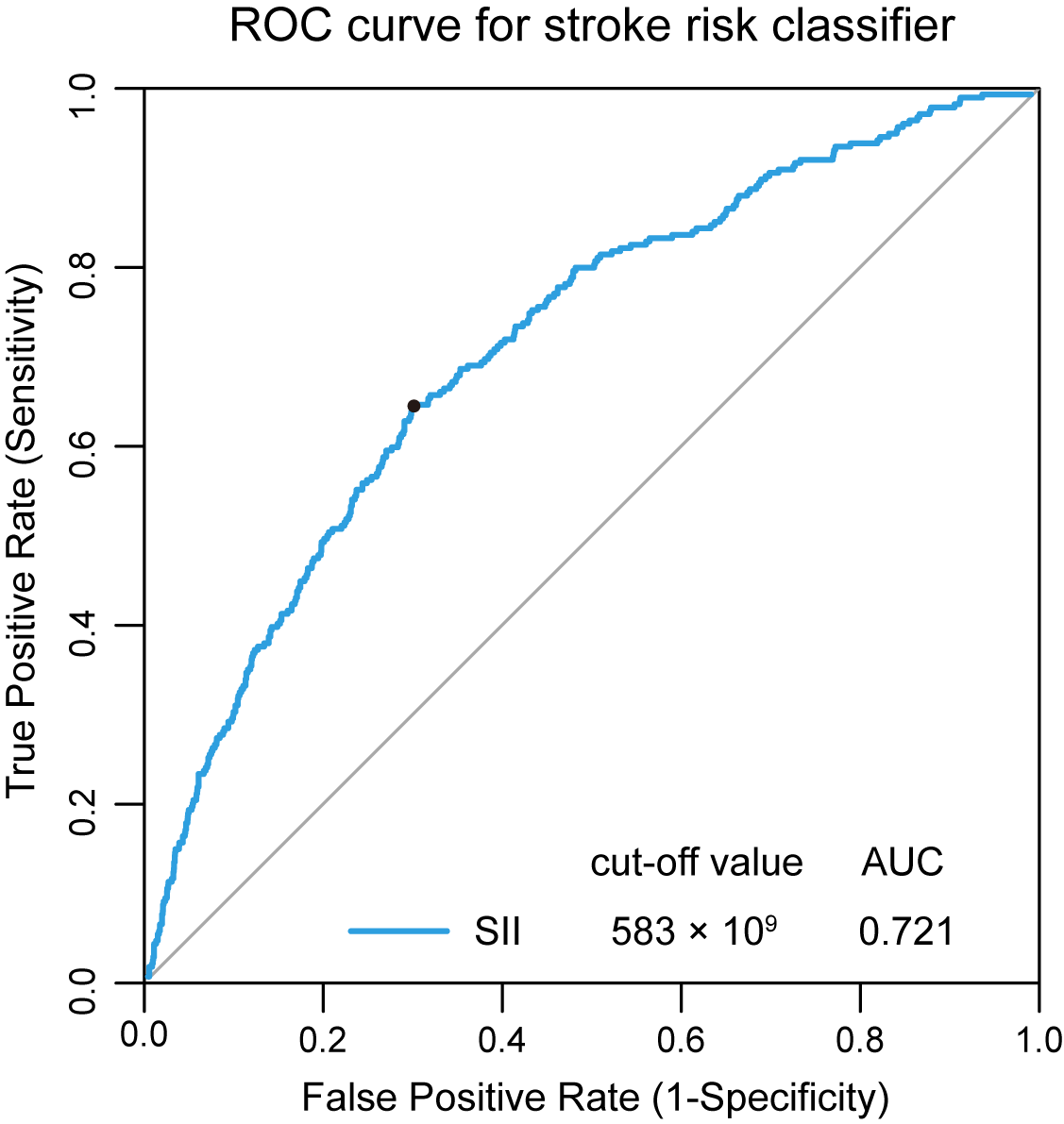
**

**Supplementary Figure 3**. ROC curve of SII for perioperative ischemic stroke. ROC, receiver operating characteristics curve; SII, systemic-immune-inflammation index; AUC, area under curve.

**Supplementary Table 2** Association between SII as continuous variable and perioperative ischemic stroke.

| Variables | Univariate analysis | | Multivariate analysis | |
| --- | --- | --- | --- | --- |
|  | OR (95% CI) | *P* value | OR (95% CI) | *P* value |
| Preoperative SII | 1.285 (1.174–1.542) | < 0.001 | 1.213 (1.169–1.468) | < 0.001 |
| Age | 1.033 (1.008–1.058) | 0.009 | 1.035 (1.006–1.064) | 0.015 |
| Sex (male vs female) | 1.092 (0.845–1.410) | 0.499 |  |  |
| BMI | 1.021 (0.986–1.058) | 0.235 |  |  |
| ASA classification |  |  |  |  |
| Class Ⅰ | reference |  |  |  |
| Class Ⅱ | 0.933 (0.425–2.634) | 0.879 |  |  |
| Class Ⅲ | 1.901 (0.852–5.412) | 0.164 |  |  |
| Hypertension (Yes vs No) | 2.184 (1.689–2.833) | < 0.001 | 1.167 (0.867–1.572) | 0.309 |
| Diabetes mellitus (Yes vs No) | 1.910 (1.454–2.491) | < 0.001 | 1.517 (1.137–2.011) | 0.004 |
| Prior ischemic stroke (Yes vs No) | 3.538 (2.480–5.166) | < 0.001 | 2.329 (1.850–3.906) | < 0.001 |
| Coronary heart disease (Yes vs No) | 1.925 (1.360–2.662) | < 0.001 | 1.099 (0.748–1.582) | 0.622 |
| Arterial fibrillation or VHD (Yes vs No) | 3.295 (1.736–5.660) | < 0.001 | 2.044 (1.021–3.747) | 0.030 |
| Peripheral vascular disease (Yes vs No) | 3.273 (2.347–4.472) | < 0.001 | 1.218 (0.804–1.809) | 0.339 |
| Renal dysfunction (Yes vs No) | 1.830 (0.719–3.777) | 0.146 |  |  |
| β-blockers medication (Yes vs No) | 2.006 (1.362–2.860) | < 0.001 | 1.074 (0.704–1.593) | 0.730 |
| Aspirin medication (Yes vs No) | 3.423 (1.921–4.341) | < 0.001 | 2.788 (2.201–3.758) | < 0.001 |
| Preoperative hemoglobin | 0.999 (0.992–1.007) | 0.888 |  |  |
| Preoperative albumin | 0.972 (0.942–1.003) | 0.077 |  |  |
| Preoperative total bilirubin | 0.998 (0.993–1.002) | 0.419 |  |  |
| Preoperative prothrombin time | 1.075 (0.971–1.150) | 0.084 |  |  |
| Preoperative MAP | 1.029 (1.017–1.041) | < 0.001 | 1.019 (1.007–1.032) | 0.003 |
| Surgical procedures |  |  |  |  |
| Trauma surgery | reference |  | reference |  |
| Spine | 2.175 (0.854–7.346) | 0.146 | 2.559 (1.334–3.374) | 0.022 |
| Intra-abdominal surgery | 0.908 (0.371–3.007) | 0.853 | 1.538 (0.594–3.263) | 0.427 |
| Joint arthroplasty | 1.636 (0.644–5.517) | 0.356 | 1.986 (1.131–4.320) | 0.047 |
| Urologic or gynecologic | 1.208 (0.719–1.963) | 0.458 | 2.075 (0.724–5.493) | 0.209 |
| Neurosurgery | 2.391 (1.445–4.708) | < 0.001 | 3.992 (2.421–5.069) | < 0.001 |
| Thoracic or vascular | 1.056 (0.592–1.788) | 0.846 | 1.048 (0.292–4.271) | 0.944 |
| Other (plastic surgery, etc) | 1.333 (0.800–2.152) | 0.252 | 2.037 (0.912–3.853) | 0.057 |
| Duration of procedures | 1.003 (1.002–1.004) | < 0.001 | 1.001 (0.999–1.003) | 0.156 |
| Estimated blood loss | 1.003 (1.001–1.005) | 0.028 | 1.003 (0.995–1.008) | 0.454 |
| MAP ≤ 65 mmHg (Yes vs No) | 1.615 (1.249–2.093) | < 0.001 | 1.372 (1.038–1.818) | 0.027 |
| Crystalloid infusion | 0.932 (0.898–0.965) | < 0.001 | 0.961 (0.919–1.002) | 0.073 |
| Colloid infusion | 1.039 (0.985–1.094) | 0.154 |  |  |
| Blood transfusion (Yes vs No) | 1.228 (0.871–1.693) | 0.224 |  |  |
| NSAIDs (Yes vs No) | 1.657 (1.215–2.307) | 0.002 | 1.237 (0.860–1.804) | 0.259 |
| Glucocorticoid (Yes vs No) | 1.369 (0.961–2.014) | 0.095 |  |  |
| Opioid dose | 1.000 (0.998–1.003) | 0.797 |  |  |
| Volatile anesthetic (Yes vs No) | 1.253 (0.746–2.309) | 0.431 |  |  |
| Preoperative NLR (< 3 vs ≥ 3) | 1.045 (1.029–1.059) | < 0.001 | 1.030 (0.998–1.056) | 0.065 |
| Preoperative PLR (< 119 vs ≥ 119) | 1.002 (1.001–1.002) | < 0.001 | 0.999 (0.998–1.001) | 0.671 |
| Abbreviations: SII, systemic-immune-inflammation index; PS, propensity score; BMI, body mass index; ASA, American Society of Anesthesiologists; VHD, valvular heart disease; MAP, mean arterial pressure; NSAIDs, nonsteroid anti-inflammatory drugs; NLR, neutrophil-lymphocyte ratio; PLR, platelet-to-lymphocyte ratio. | | | | |

**Supplementary Table 3** Univariate and multivariate logistic regression analyses for perioperative ischemic stroke in the Model 4.

| Variables | Univariate analysis | | Multivariate analysis | |
| --- | --- | --- | --- | --- |
|  | OR (95% CI) | *P* value | OR (95% CI) | *P* value |
| Preoperative SII (< 583 vs ≥ 583) | 2.217 (1.714–2.863) | < 0.001 | 1.843 (1.369–2.480) | < 0.001 |
| Age | 1.033 (1.008–1.058) | 0.009 | 1.034 (1.006–1.063) | 0.017 |
| Sex (male vs female) | 1.092 (0.845–1.410) | 0.499 |  |  |
| BMI | 1.021 (0.986–1.058) | 0.235 |  |  |
| ASA classification |  |  |  |  |
| Class Ⅰ | reference |  |  |  |
| Class Ⅱ | 0.933 (0.425–2.634) | 0.879 |  |  |
| Class Ⅲ | 1.901 (0.852–5.412) | 0.164 |  |  |
| Hypertension (Yes vs No) | 2.184 (1.689–2.833) | < 0.001 | 1.170 (0.869–1.576) | 0.301 |
| Diabetes mellitus (Yes vs No) | 1.910 (1.454–2.491) | < 0.001 | 1.521 (1.139–2.015) | 0.004 |
| Prior ischemic stroke (Yes vs No) | 3.538 (2.480–5.166) | < 0.001 | 2.358 (1.172–3.945) | < 0.001 |
| Coronary heart disease (Yes vs No) | 1.925 (1.360–2.662) | < 0.001 | 1.082 (0.736–1.559) | 0.681 |
| Arterial fibrillation or VHD (Yes vs No) | 3.295 (1.736–5.660) | < 0.001 | 2.043 (1.022–3.741) | 0.030 |
| Peripheral vascular disease (Yes vs No) | 3.273 (2.347–4.472) | < 0.001 | 1.204 (0.794–1.788) | 0.369 |
| Renal dysfunction (Yes vs No) | 1.830 (0.719–3.777) | 0.146 |  |  |
| β-blockers medication (Yes vs No) | 2.006 (1.362–2.860) | < 0.001 | 1.069 (0.700–1.586) | 0.750 |
| Aspirin medication (Yes vs No) | 3.423 (1.921–4.341) | < 0.001 | 3.405 (2.429–4.759) | < 0.001 |
| Preoperative hemoglobin | 0.999 (0.992–1.007) | 0.888 |  |  |
| Preoperative albumin | 0.972 (0.942–1.003) | 0.077 |  |  |
| Preoperative total bilirubin | 0.998 (0.993–1.002) | 0.419 |  |  |
| Preoperative prothrombin time | 1.075 (0.971–1.150) | 0.084 |  |  |
| Preoperative MAP | 1.029 (1.017–1.041) | < 0.001 | 1.019 (1.007–1.032) | 0.003 |
| Surgical procedures |  |  |  |  |
| Trauma surgery | reference |  | reference |  |
| Spine | 2.175 (0.854–7.346) | 0.146 | 3.503 (1.315–5.164) | 0.023 |
| Intra-abdominal surgery | 0.908 (0.371–3.007) | 0.853 | 1.520 (0.587–5.198) | 0.441 |
| Joint arthroplasty | 1.636 (0.644–5.517) | 0.356 | 2.905 (1.102–4.027) | 0.052 |
| Urologic or gynecologic | 1.208 (0.719–1.963) | 0.458 | 2.045 (0.714–3.380) | 0.218 |
| Neurosurgery | 2.391 (1.445–4.708) | < 0.001 | 3.167 (2.484–5.696) | < 0.001 |
| Thoracic or vascular | 1.056 (0.592–1.788) | 0.846 | 1.394 (0.442–5.293) | 0.590 |
| Other (plastic surgery, etc) | 1.333 (0.800–2.152) | 0.252 | 2.106 (0.930–4.116) | 0.063 |
| Duration of procedures | 1.003 (1.002–1.004) | < 0.001 | 1.001 (0.999–1.003) | 0.186 |
| Estimated blood loss | 1.003 (1.001–1.005) | 0.028 | 0.998 (0.994–1.001) | 0.446 |
| MAP ≤ 65 mmHg (Yes vs No) | 1.615 (1.249–2.093) | < 0.001 | 1.395 (1.056–1.848) | 0.020 |
| Crystalloid infusion | 0.932 (0.898–0.965) | < 0.001 | 0.960 (0.919–1.002) | 0.068 |
| Colloid infusion | 1.039 (0.985–1.094) | 0.154 | 1.070 (1.006–1.134) | 0.026 |
| Blood transfusion (Yes vs No) | 1.228 (0.871–1.693) | 0.224 |  |  |
| NSAIDs (Yes vs No) | 1.657 (1.215–2.307) | 0.002 | 1.239 (0.861–1.807) | 0.256 |
| Glucocorticoid (Yes vs No) | 1.369 (0.961–2.014) | 0.095 |  |  |
| Opioid dose | 1.000 (0.998–1.003) | 0.797 |  |  |
| Volatile anesthetic (Yes vs No) | 1.253 (0.746–2.309) | 0.431 |  |  |
| Preoperative NLR (< 3 vs ≥ 3) | 1.045 (1.029–1.059) | < 0.001 | 1.036 (1.007–1.062) | 0.007 |
| Preoperative PLR (< 119 vs ≥ 119) | 1.002 (1.001–1.002) | < 0.001 | 0.999 (0.998–1.001) | 0.859 |
| Abbreviations: SII, systemic-immune-inflammation index; BMI, body mass index; ASA, American Society of Anesthesiologists; VHD, valvular heart disease; MAP, mean arterial pressure; NSAIDs, nonsteroid anti-inflammatory drugs; NLR, neutrophil-lymphocyte ratio; PLR, platelet-to-lymphocyte ratio. | | | | |

**Supplementary Table 4** Univariate and multivariate logistic regression analyses for perioperative ischemic stroke in the PS matching.

| Variables | Univariate analysis | | Multivariate analysis | |
| --- | --- | --- | --- | --- |
|  | OR (95% CI) | *P* value | OR (95% CI) | *P* value |
| Preoperative SII (< 583 vs ≥ 583) | 2.195 (1.574–3.106) | < 0.001 | 1.818 (1.254–2.674) | 0.002 |
| Age | 1.023 (0.992–1.053) | 0.140 |  |  |
| Sex (male vs female) | 1.141 (0.829–1.564) | 0.416 |  |  |
| BMI | 1.024 (0.980–1.069) | 0.279 |  |  |
| ASA classification |  |  |  |  |
| Class Ⅰ | reference |  |  |  |
| Class Ⅱ | 0.968 (0.362–3.947) | 0.956 |  |  |
| Class Ⅲ | 1.872 (0.690–7.693) | 0.291 |  |  |
| Hypertension (Yes vs No) | 2.907 (2.104–4.054) | < 0.001 | 1.460 (1.001–2.142) | 0.051 |
| Diabetes mellitus (Yes vs No) | 2.229 (1.605–3.073) | < 0.001 | 1.720 (1.207–2.434) | 0.002 |
| Prior ischemic stroke (Yes vs No) | 3.504 (1.810–5.154) | < 0.001 | 2.596 (1.120–4.713) | < 0.001 |
| Coronary heart disease (Yes vs No) | 2.282 (1.519–3.329) | < 0.001 | 1.227 (0.774–1.896) | 0.370 |
| Arterial fibrillation or VHD (Yes vs No) | 2.327 (1.116–3.868) | < 0.001 | 2.533 (1.145–5.077) | 0.014 |
| Peripheral vascular disease (Yes vs No) | 3.073 (1.781–3.832) | < 0.001 | 1.522 (0.934–2.420) | 0.083 |
| Renal dysfunction (Yes vs No) | 2.489 (0.972–5.192) | 0.080 |  |  |
| β-blockers medication (Yes vs No) | 2.297 (1.460–3.480) | < 0.001 | 1.137 (0.687–1.817) | 0.603 |
| Aspirin medication (Yes vs No) | 3.884 (1.968–5.341) | < 0.001 | 3.406 (2.004–4.614) | < 0.001 |
| Preoperative hemoglobin | 1.001 (0.992–1.010) | 0.817 |  |  |
| Preoperative albumin | 0.974 (0.937–1.013) | 0.187 |  |  |
| Preoperative total bilirubin | 0.998 (0.993–1.001) | 0.325 |  |  |
| Preoperative prothrombin time | 1.053 (0.918–1.151) | 0.367 |  |  |
| Preoperative MAP | 1.032 (1.017–1.047) | < 0.001 | 1.021 (1.005–1.037) | 0.009 |
| Surgical procedures |  |  |  |  |
| Trauma surgery | reference |  | reference |  |
| Spine | 1.290 (0.430–4.716) | 0.668 | 1.258 (0.402–4.749) | 0.709 |
| Intra-abdominal surgery | 0.827 (0.333–2.757) | 0.717 | 1.104 (0.412–3.857) | 0.859 |
| Joint arthroplasty | 1.413 (0.511–4.968) | 0.540 | 1.578 (0.546–5.719) | 0.435 |
| Urologic or gynecologic | 1.049 (0.370–3.731) | 0.934 | 1.203 (0.370–4.644) | 0.769 |
| Neurosurgery | 2.791 (1.556–4.257) | < 0.001 | 3.565 (2.424–4.114) | < 0.001 |
| Thoracic or vascular | 0.797 (0.266–2.913) | 0.703 | 0.409 (0.077–1.948) | 0.258 |
| Other (plastic surgery, etc) | 1.411 (0.520–4.916) | 0.537 | 1.759 (0.510–3.387) | 0.205 |
| Duration of procedures | 1.002 (1.001–1.004) | 0.002 | 1.000 (0.998–1.002) | 0.840 |
| Estimated blood loss | 1.001 (0.997–1.005) | 0.415 |  |  |
| MAP ≤ 65 mmHg (Yes vs No) | 1.583 (1.153–2.187) | 0.005 | 1.395 (0.999–1.848) | 0.051 |
| Crystalloid infusion | 0.918 (0.877–0.959) | < 0.001 | 0.934 (0.883–0.985) | 0.014 |
| Colloid infusion | 1.018 (0.951–1.085) | 0.596 |  |  |
| Blood transfusion (Yes vs No) | 1.141 (0.747–1.685) | 0.523 |  |  |
| NSAIDs (Yes vs No) | 1.651 (1.129–2.491) | 0.013 | 1.339 (0.857–2.137) | 0.209 |
| Glucocorticoid (Yes vs No) | 1.305 (0.849–2.016) | 0.248 |  |  |
| Opioid dose | 1.000 (0.997–1.003) | 0.875 |  |  |
| Volatile anesthetic (Yes vs No) | 1.794 (0.865–4.573) | 0.161 |  |  |
| Preoperative NLR (< 3 vs ≥ 3) | 1.042 (1.024–1.057) | < 0.001 | 1.035 (1.002–1.063) | 0.020 |
| Preoperative PLR (< 119 vs ≥ 119) | 1.003 (1.001–1.005) | < 0.001 | 0.999 (0.998–1.001) | 0.920 |
| Abbreviations: SII, systemic-immune-inflammation index; PS, propensity score; BMI, body mass index; ASA, American Society of Anesthesiologists; VHD, valvular heart disease; MAP, mean arterial pressure; NSAIDs, nonsteroid anti-inflammatory drugs; NLR, neutrophil-lymphocyte ratio; PLR, platelet-to-lymphocyte ratio. | | | | |
